# Maternal nutrient restriction in mid-to-late gestation influences fetal mRNA expression in muscle tissues in beef cattle

**DOI:** 10.1186/s12864-017-4051-5

**Published:** 2017-08-18

**Authors:** Francois Paradis, Katie M. Wood, Kendall C. Swanson, Stephen P. Miller, Brian W. McBride, Carolyn Fitzsimmons

**Affiliations:** 1 Department of Agriculture, Food, and Nutritional Science, University of Alberta, 4-10 Agriculture-Forestry Centre, Edmonton, AB T6G 2P5 Canada; 2 Department of Animal Biosciences, University of Guelph, Guelph, ON N1G 2W1 Canada; 3 Department of Animal Science, North Dakota State University, Fargo, ND 58102 USA; 4 Angus Genetic Inc., St. Joseph, MO 64506 USA; 5 Agriculture and Agri-Food Canada, Edmonton, AB T6G 2C8 Canada

**Keywords:** Beef cattle, fetal programming, nutrient restriction, liver, muscle, *IGF2*, methylation, microRNA

## Abstract

**Background:**

Manipulating maternal nutrition during specific periods of gestation can result in re-programming of fetal and post-natal development. In this experiment we investigated how a feed restriction of 85% compared with 140% of total metabolizable energy requirements, fed to cows during mid-to-late gestation, influences phenotypic development of fetuses and mRNA expression of growth (Insulin-Like Growth Factor family and Insulin Receptor (*INSR*)), myogenic (Myogenic Differentiation 1 (*MYOD1*), Myogenin (*MYOG*), Myocyte Enhancer Factor 2A (*MEF2A*), Serum Response Factor (*SRF*)) and adipogenic (Peroxisome Proliferator Activated Receptor Gamma (*PPARG*)) genes in fetal *longissimus dorsi* (LD) and *semitendinosus* (ST) muscle. DNA methylation of imprinted genes, Insulin Like Growth Factor 2 (*IGF2*) and Insulin Like Growth Factor 2 Receptor (*IGF2R*), and micro RNA (miRNA) expression, were also examined as potential consequences of poor maternal nutrition, but also potential regulators of altered gene expression patterns.

**Results:**

While the nutrient restriction impacted dam body weight, no differences were observed in phenotypic fetal measurements (weight, crown-rump length, or thorax circumference). Interestingly, LD and ST muscles responded differently to the differential pre-natal nutrient levels. While LD muscle of restricted fetal calves had greater mRNA abundances for Insulin Like Growth Factor 1 and its receptor (*IGF1* and *IGF1R*), *IGF2R*, *INSR*, *MYOD1*, *MYOG*, and *PPARG*, no significant differences were observed for gene expression in ST muscle. Similarly, feed restriction had a greater impact on the methylation level of *IGF2* Differentially Methylated Region 2 (DMR2) in LD muscle as compared to ST muscle between treatment groups. A negative correlation existed between *IGF2* mRNA expression and *IGF2* DMR2 methylation level in both LD and ST muscles. Differential expression of miRNAs 1 and 133a were also detected in LD muscle.

**Conclusions:**

Our data suggests that a nutrient restriction of 85% as compared to 140% of total metabolizable energy requirements during the 2nd half of gestation can alter the expression of growth, myogenic and adipogenic genes in fetal muscle without apparent differences in fetal phenotype. It also appears that the impact of feed restriction varies between muscles suggesting a priority for nutrient partitioning depending on muscle function and/or fiber composition. Differences in the methylation level in *IGF2*, a well-known imprinted gene, as well as differences in miRNA expression, may be functional mechanisms that precede the differences in gene expression observed, and could lead to trans-generational epigenetic programming.

## Background

Maternal nutritional stimuli or insult during gestation can lead to genetic and phenotypic changes on the developing fetus that may also affect post-natal development of the calf [1]. Fetal developmental programming can have implications for the beef industry, as the impacts of these stimuli may influence fertility, health and immunity, growth potential, carcass yield, and overall muscle mass and composition, all of which impacts profitability [2].

Previous work in cattle and other ruminants have often focused on nutritional insults in early-to-mid gestation [3–5]. However, during mid-to-late gestation the fetus undergoes rapid growth, representing a more significant metabolic toll on the dam [6]. Vital organs such as brain and heart, and to some extent liver, remain relatively immune to the effects of prenatal maternal nutrition during the 1st trimester of fetal development in the bovine [7, 8], and appear to have priority over skeletal muscle for nutrient partitioning. Therefore, since growth predominates during the latter half of gestation, and is of a lower priority for nutrient partitioning in the fetus, sub-optimal maternal nutrition at this stage could be detrimental for fetal growth and muscle development [2, 9]. Previous research has shown that heifers born from cows receiving supplementation in the last trimester of gestation were heavier at weaning, pre-breeding and at pregnancy confirmation compared to unsupplemented cows [10], and that steers born from dams managed on a higher plane of nutrition (improved pasture) as compared to a low plane of nutrition (native range), had improved meat tenderness characteristics, animal growth, and carcass composition [11]. Therefore proper late gestation nutrition can support fetal programming events towards improved growth and meat quality traits.

Fetal muscle development has been shown to be sensitive to nutrient abundance. The number, type and size of muscle fibres are the main determinant of muscle mass and total fibre number is positively correlated to growth potential [12, 13]. Interestingly, while muscle fibre hypertrophy occurs mostly after birth, muscle fibre number and type is determined in utero ([14], and as reviewed by [7]). In cattle fetuses, by day 240 of gestation, total muscle fibre number has been fixed consequently predetermining the lifetime potential of an animal [13]. Moreover, other meat characteristics such as marbling can be impacted prenatally [9]. Changes in offspring total muscle fibre number and/or in muscle fibre type have been observed as a consequence of maternal under- and over-nutrition in ewes, sows and rats [5, 15–17]. Therefore nutritional restriction has the potential to hamper the performance and muscle development of its offspring

Muscle development in utero is a complex process involving proliferation and differentiation of the myogenic precursor cells [12]. IGF2 is a potent mitogen and inducer of cell differentiation known to impact fetal growth and muscle development [13]. It is known to be transcribed from at least 4 different promoters, generating several transcript variants, each possessing their own spatio-temporal expression signature during development and growth [18]. It is also a well-known imprinted gene in several species and interestingly, its methylation status has been found to be affected by prenatal nutrition in humans [19]. The latter is particularly important because changes in DNA methylation will not only affect the tissue composition of the animal but it has a greater chance to be inherited by subsequent generations affecting their offspring phenotype.

A group of myogenic regulatory factors (MRF) including MYOD1 and MYOG are important transcription factors involved in myogenesis [12]. While MYOD1 has been shown to be important for formation and survival of myoblasts, MYOG and MYOD1 have also been shown to be involved in terminal differentiation of myotubes. Any impacts upon muscle development in utero may in concert affect the expression of these MRF.

Understanding the molecular mechanisms underlying the differences in fetal skeletal muscle development in response to maternal nutrition is of considerable practical significance. Therefore, the objectives of this experiment were to determine the consequences of moderate maternal nutrient restriction during the 2nd half of gestation: 1) on fetal phenotypic characteristics, 2) on the expression of growth, myogenic and adipogenic genes and miRNA in the fetal muscles and 3) on DNA methylation levels in the imprinted genes *IGF2* and *IGF2R* in two muscle types.

## Methods

### Animals, experimental design and dietary treatments

This experiment was conducted in accordance with the guidelines from the Canadian Council on Animal Care and with the approval of the University of Guelph Animal Care Committee. The experiment was a continuation of a study investigating maternal metabolism in mid-to-late gestation described previously by Wood et al. [20]. Briefly, 24 mature (3–6 yrs. old) multiparous pregnant Angus-Simmental cross-bred cows were used in a randomized complete block design where cows were blocked (*n* = 6) by expected date of parturition. The cows were divided into 2 dietary groups (*n* = 12 cows/diet), and fed one of 2 levels of nutrition; 1) ad-libitum intake (~140% of metabolizable energy requirements [21, 22]; HIGH), and 2) restricted to 85% of metabolizable energy requirements (LOW), as calculated from dry matter intake (DMI) of the cows described by Wood et al. [20]. The two levels of energy supplied to the cattle were chosen to represent a moderate energy restriction verses the energy intake a cattle producer would realize if cows were fed ad libitum on the same diet as used in this study. The diet consisted of a haylage-based total mixed ration containing 20% wheat straw and contained a commercially available trace mineral and vitamin supplement [20]. All cattle started the feeding trial simultaneously at 147 ± 15 days of gestation and were slaughtered over 6 weeks (blocks), in groups of 4 (2 cows from both groups/week) at 247 ± 10 days of gestation. DMI was measured for individual animals using Calan gates (American Calan, Inc., Northwood, NH). Intakes were adjusted every 14 d based on body weight.

#### Sample Collection and Carcass Measurements

The pregnant cows were slaughtered using captive-bolt stunning and exsanguination at the University of Guelph Meat Laboratory, and fetal phenotypic evaluation was performed. Fetal weight, crown-rump length and thorax circumference were measured immediately after obtaining the fetuses. Fetal organs including heart, liver, placenta and kidney as well as the cow uterus were dissected free of connective tissue and weighed. The placenta and uterus were then further dissected to isolate the caruncles and cotyledons. The fetal LD and ST muscles were also dissected free of connective and fat tissue. Two samples of approximately 5 g of heart, liver, caruncle, cotyledon, LD and ST muscles were collected, rinsed in ice-cold saline, snap frozen in liquid nitrogen and stored at −80 °C. Two cows, one from both the HIGH and LOW treatments, were carrying twin fetuses. Due to potential differences in nutrient demand from twins as opposed to singleton pregnancies, data from these cows and fetuses were removed leaving the number of individuals equal to eleven for both treatment groups for all subsequent analyses.

#### RNA extraction and real-time PCR

Heart, liver, caruncle, cotyledon, and LD and ST muscle samples were ground under liquid nitrogen using a mortar and pestle. Total RNA was extracted from 30 to 50 mg of heart, caruncle and cotyledon using TRIzol reagent (Life technologies) following the manufacturer’s instructions. The total RNA was precipitated with 0.5 volume of isopropanol and resuspended in nuclease-free H_2_O (Ambion, Foster City, CA, USA). Total RNA from liver, LD and ST muscle was extracted from 30 to 50 mg of tissue using the miRNeasy micro Kit (Qiagen, Germantown, MD, USA) according to manufacturer’s instructions. All total RNA samples were quantified using a spectrophotometer ND-1000 (NanoDrop, Wilmington, DE, USA), evaluated for RNA integrity (RIN) using an Agilent 2100 Bioanalyser (Agilent Technologies, Santa Clara, CA, USA) and stored at -80 °C until cDNA synthesis. The RIN value of RNAs isolated from all tissues were >7, with the exception of fetal liver and maternal caruncle, which were >6.5.

### Gene expression analysis

Total RNA (2 μg) from each individual calf and tissue was reverse transcribed with the High Capacity cDNA Reverse Transcription Kit (Life Technologies Inc., Carlsbad, CA, USA) according to the manufacturer’s instructions. RNaseOUT (Life Technologies Inc.) was also added to the reaction at a concentration of 2 U/μl. After reverse transcription (RT), the cDNA were diluted to 0.5 ng/μl with nuclease-free H_2_O (Ambion). Real-Time PCR analysis was performed in duplicate using 1 ng of cDNA in 96-well fast plates using the SYBR fast master mix ABI prism (D-Mark Biosciences) and the Step-One Plus Real-time PCR system (Life Technologies Inc.). A blank sample and a minus RT were added to control for nonspecific amplification. Relative standard curves, made from a serial dilution of pooled cDNA from the tissue of interest and ranging from 20 to 0.02 ng, were used to determine the relative quantity of each sample. The primers were designed in Primer3 [23–25] using species-specific sequences found in GenBank, were designed to cover exon-exon junctions when possible, and ran with a annealing/extension temperature in the real-time PCR reaction of 60 °C (Table 1). The amplification efficiency for each gene was determined using serial dilution of tissue specific cDNA and was found to be 100 ± 10% for all genes (data not shown). The resulting quantitative PCR (qPCR) amplicon were also sequenced to confirm their identity (data not shown). For each tissue, 2–4 endogenous controls were tested and the best individual or combination of endogenous control was chosen using NormFinder [26]. Therefore, Peptidylprolyl Isomerase A (*PPIA*), Hydroxymethylbilane Synthase (*HMBS*), Eukaryotic Translation Elongation Factor 1 Alpha 2, (*EEF1A2*), Tyrosine 3-Monooxygenase/Tryptophan (*YWHAZ*) and the geometric mean of *YWHAZ/*Glyceraldehyde-3-Phosphate Dehydrogenase (*GAPDH*) were used as the endogenous control to correct for RNA extraction and reverse transcription efficiency in liver, heart, muscle (LD and ST), cotyledon, and caruncle, respectively. The endogenous controls were also tested for any treatment effect and were found to be stable among samples within each tissue type confirming their usefulness as suitable endogenous controls. For all genes only the expression of transcripts and miRNAs that could be reliably detected in each tissue type are reported.

**Table 1:** Primer sequences and amplification conditions for gene expression measured by real-time PCR

Gene	GenBank Accession #	Primer	Sequence 5′ - 3’	Product size (bp)
*ACTB*	NM_173979.3	Fwd	CTCTTCCAGCCTTCCTTCCT	245
		Rev	CCAATCCACACGGAGTACTTG	
*EEF1A2*	NM_001037464.1	Fwd	AGTTCACGTCCCAGGTCATC	149
		Rev	CTCCAACTTCTTGCCAGAGC	
*FLT1*	NM_001191132.2	Fwd	AGGGAAGAAGGTGGTCATCC	185
		Rev	TGACTGTTGTCTCGCAGGTC	
*GAPDH*	NM_001034034.1	Fwd	TGACCCCTTCATTGACCTTC	143
		Rev	GATCTCGCTCCTGGAAGATG	
*HMBS*	NM_001046207.1	Fwd	CTACTTCGCTGCATTGCTGA	105
		Rev	CAGGTACAGTTGCCCATCCT	
*IGF1*	NM_001077828	Fwd	GATGCTCTCCAGTTCGTGTG	141
		Rev	CTCCAGCCTCCTCAGATCAC	
*IGF1R*	NM_001244612	Fwd	CAAAGGCAATCTGCTCATCA	139
		Rev	CAGGAAGGACAAGGAGACCA	
*IGF2*	NM_174087.3	Fwd	CCAGCGATTAGAAGTGAGCC	95
		Rev	AGACCTAGTGGGGCGGTC	
*IGF2R* ^a^	NM_174352	Fwd	GCAATGCTAAGCTTTCGTATTACG	188
		Rev	GGTGTACCACCGGAAGTTGTATG	
*INSR*	XM_002688832	Fwd	CCTATGCCCTGGTGTCACTT	114
		Rev	GCTGCCTTAGGTTCTGGTTG	
*KDR*	NM_001110000.1	Fwd	ATCGAAGTTTCCTGCACAGC	133
		Rev	TCACCCTGCGGATAGTTAGG	
*MEF2A*	NM_001083638	Fwd	CAATGCCAACTGCCTACAAC	130
		Rev	TGTCCTAAATGGTGCTGCTG	
*MYOD1*	NM_001040478	Fwd	GAACACTACAGCGGCGACTC	121
		Rev	AGTAAGTGCGGTCGTAGCAG	
*MYOG*	NM_001111325	Fwd	CAGTGAATGCAGCTCCCATA	164
		Rev	CGACATCCTCCACTGTGATG	
*PGF*	NM_173950	Fwd	GGAACATTTCATCGGAGGTG	136
		Rev	CAGGATGGGCTGAATAGATG	
*PPARG*	NM_181024,2	Fwd	ACCACCGTTGACTTCTCCAG	137
		Rev	ACAGGCTCCACTTTGATTGC	
*PPIA*	NM_178320.2	Fwd	GTCAACCCCACCGTGTTCT	132
		Rev	TCCTTTCTCTCCAGTGCTCAG	
*SLC2A1*	NM_174602.2	Fwd	ACACAGCCTTCACTGTCGTG	156
		Rev	TGCTCAGGTAGGACATCCAG	
*SLC2A3*	NM_174603	Fwd	CCTCTGATCTTCGCCATCTC	170
		Rev	AAGGACCACAGGGATGTGAG	
*SLC7A1*	NM_001135792.1	Fwd	AGCAGCAGCGATTCTCAGAC	158
		Rev	AGGCAGAAGGTGATGACCAG	
*SRF*	NM_001206016	Fwd	CGGCTTTGAAGAGACAGACC	101
		Rev	GCAGGTTGGTGACGGTAAAC	
*VEGFA*	NM_174216.1	Fwd	AGACCCTGGTGGACATCTTC	185
		Rev	TATGTGCTGGCTTTGGTGAG	
*YWHAZ*	NM_174814.2	Fwd	AGACGGAAGGTGCTGAGAAA	123
		Rev	CGTTGGGGATCAAGAACTTT	

^a^Spicer et al. [48]

### MicroRNA analysis

For miRNA analysis, 200 ng of total RNA from LD muscle was reverse transcribed with miRNA specific primers using the TaqMan microRNA Reverse Transcription Kit (Life Technologies Inc.) according to manufacturer’s instructions. The cDNA was then diluted to 0.25 ng/μl with nuclease-free H_2_O (Ambion). Real-time PCR for miRNA analysis in LD samples was performed in duplicate using 0.5 ng of cDNA as described previously but using the TaqMan Universal Master Mix (Life Technologies Inc.), and the miRNA specific primers. A relative standard curve ranging from 5 to 0.005 ng was also used to determine the relative quantity of each sample. Three miRNA endogenous controls (microRNA 16b (miR-16b), microRNA-191 (miR-191), and RNA, U6 Small Nuclear 6 (RNU6B)) were tested and the best individual or combination of endogenous control was chosen using NormFinder (24). The geometric mean of miR-16b and miR-191 was used to normalize the data. Amplification efficiency and endogenous control stability was also determined as described previously.

### DNA methylation analysis

#### Genomic DNA extraction

Genomic DNA (gDNA) was extracted from LD and ST muscle tissue by overnight digestion at 50 °C in digestion buffer [100 mM NaCl, 10 mM Tris, 25 mM EDTA, 0.5% (*w*/*v*) SDS, 80 μg/ml RNaseA and 0.1 mg/ml Proteinase K]. The gDNA was then extracted with Phenol:Chloroform:Isoamyl Alcohol (25:24:1 (*v*/v)) using the Maxtract Tube (Qiagen), precipitated with 0.5 volume of 7.5 M ammonium acetate and 2 volume 100% ethanol, resuspended in nuclease-free H_2_O (Ambion) and stored at -20 °C. The gDNA was then quantified using the Quant-iT PicoGreen dsDNA assay kit (Life technologies) according to manufacturer’s instructions and its integrity was evaluated on a 0.8% (*w*/*v*) agarose gel.

#### Methylation analysis

DNA methylation analysis of partial regions from the *IGF2* DMR2 (UCSC_Btau_4.6.1_Chr29:51,079,954–51,080,478), *IGF2/H19* intergenic control region (ICR) (UCSC_Btau_4.6.1_Chr29:51,165,094–51,165,593), and *IGF2R* DMR2 (UCSC_Btau_4.6.1_Chr9:100,467,361–100,467,716) was performed by McGill University and Genome Quebec Innovation Centre using EpiTYPER® DNA methylation analysis technology (Agena Bioscience) according to their standard procedure. Briefly, 1 μg of gDNA was treated with sodium bisulfite using the EZ-96 DNA Methylation Kit (Zymo Research, Irvine, CA, USA) according to the manufacturer’s instructions, except the bisulfite conversion was performed with 15 μl of sample, 5 μl of M-Dilution Buffer and 130 μl of CT-Conversion Reagent followed by 16 cycles of 95 °C for 30 s and 50 °C for 1 h. Bisulfite treated DNA was eluted from the Silicon-A Binding plate in 70 μl of M-Elution Buffer. The bisulfite treated DNA was then PCR amplified, treated with Shrimp Alkaline Phosphatase, in vitro transcribed and submitted to base-specific RNA cleavage according to the manufacturer’s instructions (Agena Bioscience). The primers used in this experiment were designed using EpiDesigner (Agena Bioscience; Table 2). The transcription cleavage products were then conditioned with CLEAN resin and transferred to a SpectroCHIP® using the MassARRAY nanodipenser (Agena Bioscience). The MALDI-TOF mass spectra were acquired using the MassARRAY analyser and the data were processed with the EpiTYPER software (Agena Bioscience). Non-quantifiable and ambiguous CpG were excluded from the analysis.

**Table 2:** Primers used to amplify each of the three DMRs investigated

Gene	Location	Primer	Sequence 5′ - 3’	Product size (bp)	Total CpG sites (Analysable site)
*IGF2R* DMR2	UCSC_Btau_4.6.1 Chr9:100,467,361–100,467,716	Fwd	aggaagagagGAGTAAAGATTTGAAATTGAAAGTAG	356	17 (11)
		Rev	cagtaatacgactcactatagggagaaggctAACCTTCTCAACACCTTACTCAAAA		
*IGF2* DMR2	UCSC_Btau_4.6.1 Chr29:51,079,954–51,080,478	Fwd	aggaagagagGTATTTTGGGTATTTGGGGTAGTTT	525	22 (10)
		Rev	cagtaatacgactcactatagggagaaggctATTCTAATCCCCTCAACCAAATAAA		
*IGF2/H19* ICR	UCSC_Btau_4.6.1 Chr29:51,165,094–51,165,593	Fwd	aggaagagagGGATAGGAGATTAGGTTTAGAGGGG	500	17 (14)
		Rev	cagtaatacgactcactatagggagaaggctAAAAAAAACTATAAAATCCTCCTACCC		

### Statistical analysis

Prior to the statistical analysis, the data from 2 cows (1 from each dietary treatment) and their corresponding fetuses were removed from the analysis because of the confounding effect of being pregnant with twins, leaving eleven fetuses/treatment group. The cow and fetal phenotypic data, the real-time PCR data and the methylation data were analysed using the MIXED procedure of SAS (SAS Institute Inc., Cary, NC). The model for the experiment included block (date of parturition) and diet as the fixed independent variables, and pen nested within block and treatment as the random variable. The interaction of fetal sex by treatment was also tested in the model but was found to be non-significant and was removed. Differences between means were analysed using a Least Significant Difference (LSD) test at a 95% confidence level. When necessary, the data were transformed to meet the assumption of normality and homoscedasticity. Correlation analyses were also performed in LD and ST muscle to investigate relationships between fetal weight and the mRNA abundance for each gene, between *IGF2* and the myogenic genes and between DNA methylation and *IGF2* and *IGF2R* expression.

## Results

### Phenotypic data

The cows used in this experiment showed no differences in body weight between treatments at the beginning of the feeding trial (Table 3; as also reported by Wood et al., [20]). During and at the end of the feeding trial HIGH cows had higher dry matter intake (DMI) and average daily gain (ADG) (*P* < 0.01), which resulted in greater total body weight gain and heavier body weight at the end of trial (*P* < 0.05, Table 3). On the other hand, at slaughter the fetuses obtained from HIGH and LOW cows showed no differences in age at slaughter, body weight, crown-rump length, thorax circumference, and organ weight (Table 3). Similarly, no differences in uterine and placental weight were observed between the HIGH and LOW diet treatments.

**Table 3:** The impact of mild nutrient restriction or no restriction during mid-to-late gestation on cow weight gain, and fetal weight and organ weight, and uterine and placental weight

Item		Treatment		*P*-value
		High diet^a^	Low diet^a^	
Cow^b^	Start weight (kg)	645.5 ± 21.8	633.4 ± 20.9	0.726
	Final weight (kg)	749.3 ± 18.7	686.3 ± 19.3	0.0438
	Weight gain (kg)	103.8 ± 7.6	52.9 ± 8.2	0.0015
	Average daily gain (kg/d)	1.06 ± 0.08	0.53 ± 0.07	0.0012
	Average dry matter intake (kg/d)	11.00 ± 0.28	6.46 ± 0.18	< 0.0001
	Uterine Weight (g)	4827.4 ± 206.7	4463.2 ± 284.1	0.338
Fetus	Age at slaughter (d)	247.8 ± 4.1	245.7 ± 1.2	0.743
	Weight	31.9 ± 1.7	28.5 ± 2.4	0.332
	Crown-rump length (cm)	78.2 ± 2.4	75.9 ± 3.8	0.696
	Thorax circumference (cm)	65.6 ± 1.9	62.2 ± 2.0	0.339
	Heart weight (g)	210.9 ± 11.9	196.7 ± 17.7	0.607
	Liver weight (g)	806.7 ± 47.0	683.6 ± 66.2	0.199
	Kidney weight (g)	89.4 ± 6.1	80.2 ± 5.9	0.253
	Placental weight (g)	3562.1 ± 297.7	3837.1 ± 362.6	0.533

^a^Data are expressed as lsmeans ± SEM

^b^Also reported in Wood et al. [20]

### Gene and miRNA expression

**Fig. 1:**
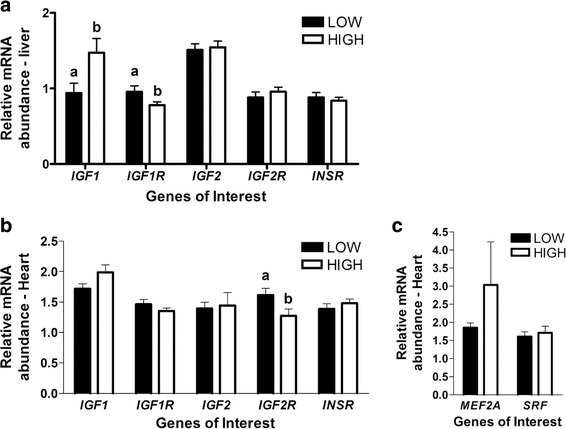
mRNA abundance for Insulin-like growth factors and myogenesis related genes in liver (**a**) and heart (**b, c**) tissue from fetal calf (*n* = 22) exposed to a high or low diet in utero during the 2nd half of gestation. Different letters represent significant differences (*P* < 0.05 ^a,b^)

Analysis of the expression of insulin-like growth factor family members in liver tissue revealed increased *IGF1* and reduced *IGF1R* mRNA abundance in fetuses from HIGH cows (*P* < 0.05, Fig. 1a). In the heart, a reduction in *IGF2R* mRNA abundance in the HIGH fetuses was observed (*P* < 0.05, Fig. 1b), while no differences in expression of myogenic genes *MEF2A* and *SRF* were observed (Fig. 1b).

**Fig. 2:**
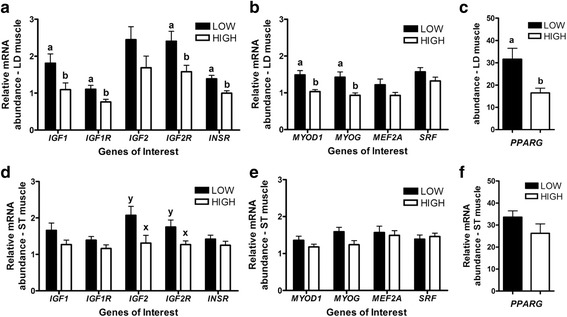
mRNA abundance for Insulin-like growth factors, and myogenesis and adipogenesis related genes, in longissimus dorsi (**a**, **b**, **c**) and semitendinosus (**d**, **e**, **f**) muscle from fetal calf (*n* = 22) exposed to a high or low diet in utero during the 2nd half of gestation. Different letters represent significant differences (*P* < 0.05 ^a,b^; *P* < 0.1^×,y^)

**Fig. 3:**
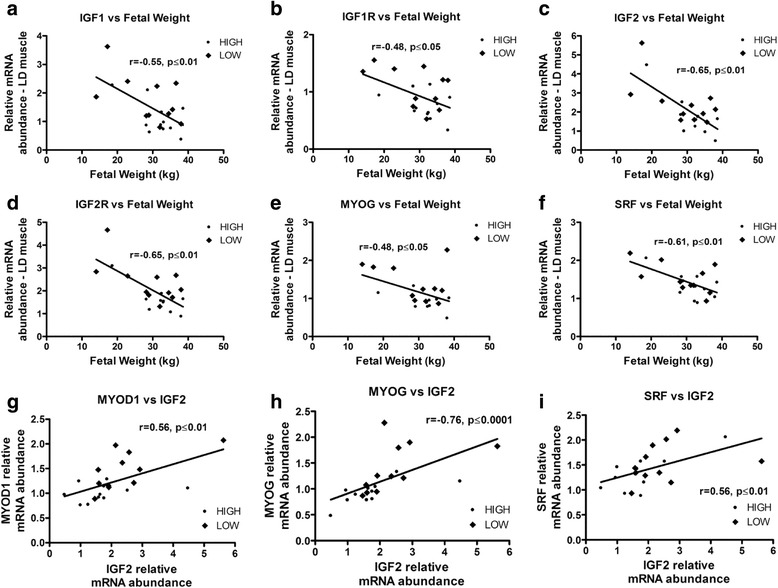
Correlation analysis between fetal weight and *IGF1* (**a**), *IGF1R* (**b**), *IGF2* (**c**), *IGF2R* (**d**), *MYOG* (**e**) and *SRF* (**f**) mRNA abundance and between *IGF2* and *MYOD1* (**g**), *MYOG* (**h**) and *SRF* (**i**) mRNA abundance in fetal LD muscle. Only significant (*P* < 0.05) correlations are reported

**Fig. 4:**
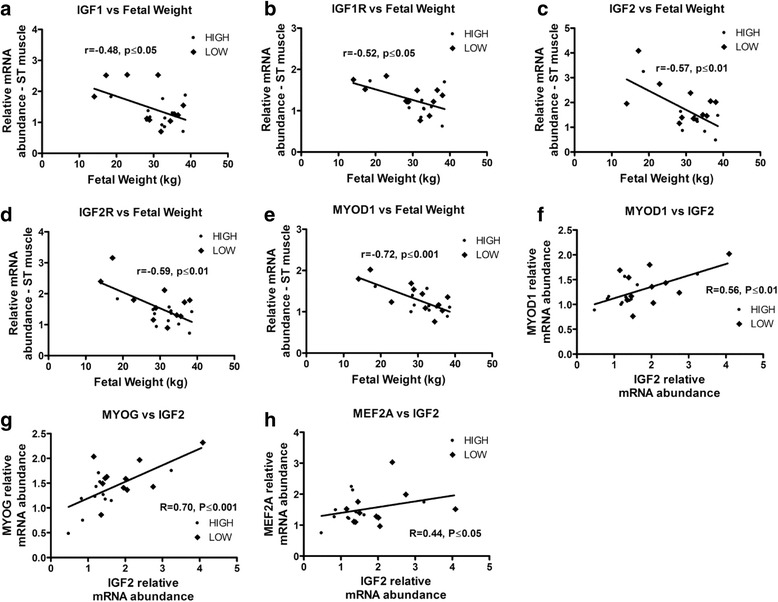
Correlation analysis between fetal weight and *IGF1* (**a)**, *IGF1R* (**b**), *IGF2* (**c**), *IGF2R* (**d**) and *MYOD1* (**e**) mRNA abundance and between *IGF2* and *MYOD1* (**f**), *MYOG* (**g**) and *MEF2A* (**h**) mRNA abundance in fetal ST muscle. Only significant (*P* < 0.05) correlations are reported

In fetal LD muscle, gene expression analysis of the insulin-like growth factors, and the myogenic and adipogenic families, revealed many differences associated with maternal HIGH and LOW diets (Fig. 2a-c). *IGF1*, *IGF1R*, *IGF2R* and *INSR* mRNA had greater abundance in the muscle from LOW fetuses (*P* < 0.05, Fig. 2a). Similarly, *MYOD1*, *MYOG* and *PPARG* mRNA also had greater abundance in the LD muscle of LOW fetuses (*P* < 0.05, Fig. 2b, c). Interestingly, LD expression of *IGF1* (*r* = −0.55, *P* ≤ 0.01), *IGF1R* (*r* = −0.48, *P* ≤ 0.05), *IGF2* (*r* = −0.65, *P* ≤ 0.01), *IGF2R* (*r* = −0.65, *P* ≤ 0.01), *MYOG* (*r* = −0.48, *P* ≤ 0.05) and *SRF* (*r* = −0.61, *P* ≤ 0.01) were negatively correlated with fetal weight (Fig. 3a-f). In addition, in LD muscle *IGF2* mRNA abundance was positively correlated with that of *MYOD1* (*r* = 0.56, *P* ≤ 0.01), *MYOG* (*r* = 0.76, *P* ≤ 0.0001), and *SRF* (*r* = 0.56, *P* ≤ 0.01) (Fig. 3g-i). The results differed in the ST muscle where only trends for increased *IGF2* and *IGF2R* mRNA abundance were observed in LOW fetuses (*P* < 0.1, Fig. 2d-f). Nonetheless, in ST negative correlations were also observed between *IGF1* (*r* = −0.48, *P* ≤ 0.05), *IGF1R* (*r* = −0.52, *P* ≤ 0.05), *IGF2* (*r* = −0.57, *P* ≤ 0.01), *IGF2R* (*r* = −0.59, *P* ≤ 0.01) and *MYOD1* (*r* = −0.72, *P* ≤ 0.001) with fetal weight (Fig. 4a-e). In ST muscle, *IGF2* mRNA abundance was positively correlated with *MYOD* (*r* = 0.56, *P* ≤ 0.01), *MYOG* (*r* = 0.70, *P* ≤ 0.001) and *MEF2A* (*r* = 0.44, *P* ≤ 0.05) (Fig. 4f-h).

**Fig. 5:**
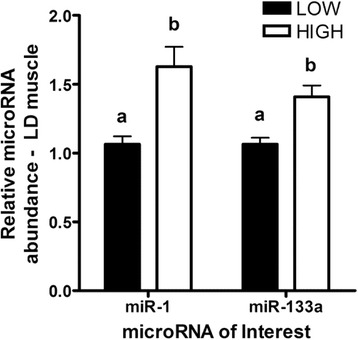
microRNA abundance for miR-1 and miR-133a in the LD muscle of fetal calves (*n* = 22) exposed to a high or low diet in utero during the 2nd half of gestation. Different letters represent significant differences (*P* < 0.05^a,b^)

**Fig. 6:**
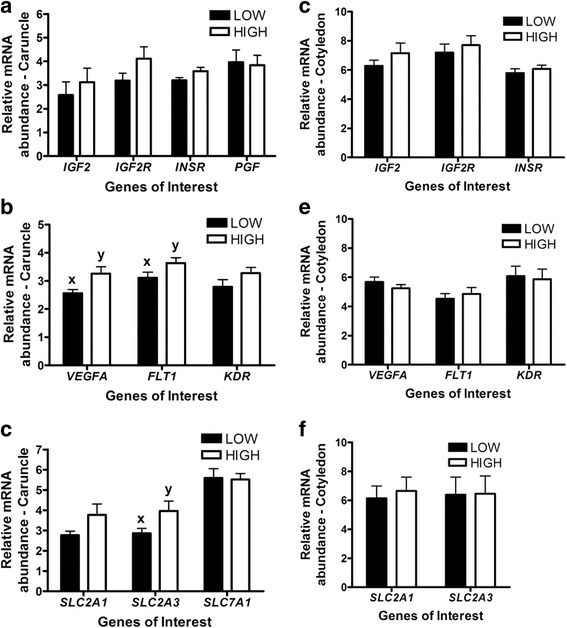
mRNA abundance for Insulin-like growth factors, angiogenesis and solute carrier related genes in the placenta (Caruncle (**a, b, c**); Cotyledon (**d, e, f**)) of fetal calves (*n* = 22) exposed to a high or low diet in utero during the 2nd half of gestation. Different letters represent differences at *P* < 0.1^x,y^

Analysis of microRNA 1 (miR-1) and microRNA 133a (miR-133a) expression also revealed that both microRNAs abundance was lower in the LD muscle of LOW fetuses (*P* < 0.05, Fig. 5). Lastly, although trends for differential mRNA abundance of the angiogenic factors Vascular Endothelial Growth Factor A (*VEGFA)* and Fms Related Tyrosine Kinase 1 (*FLT1*)*,* and the glucose transporter Solute Carrier Family 2 Member 3 (*SLC2A3*) were observed in caruncles of HIGH and LOW cows (increased in HIGH), no other significant differences were observed in the placenta (Fig. 6).

### DNA methylation

Methylation analysis of the *IGF2*/*H19* ICR in LD and ST muscle did not reveal any differences in methylation level between HIGH and LOW fetuses. Similarly, only the CpG group 23–24 of the *IGF2R* DMR was differently methylated in the LD muscle of HIGH and LOW fetuses (0.344 vs 0.363 respectively; *P* = 0.039). No correlations were observed between the CpG group 23–24 and *IGF2R* mRNA abundance. On the other hand, several CpG and CpG groups of the *IGF2* DMR2 were differentially methylated in the LD muscle of HIGH and LOW diet fetuses (Table 4). In all instances, the methylation levels of these CpG or CpG groups were higher in the muscle of HIGH fetuses and the differences in methylation level observed between the two groups varied between 2.5 and 10.1% (Table 4). Moreover, the overall methylation level for DMR2 in LD muscle was greater in the HIGH fetuses (*P* < 0.05), and while a similar trend was observed in ST muscle the difference was not significant (Table 4). Although HIGH fetuses had greater methylation levels in all quantifiable CpG in *IGF2* DMR2 in both LD and ST muscle, only in the LD muscle were the differences significant at *P* < 0.05. This observation is consistent with the observed gene expression differences between the two muscle types. The methylation level of several CpG and CpG groups in the DMR2 of *IGF2* was negatively correlated with *IGF2* mRNA abundance in both muscle tissues (Table 4). Using the web-based prediction program TFBIND (http://tfbind.hgc.jp) [27], we identified several putative transcription factor binding sites in the *IGF2* DMR2 region including potential binding sites for MYOD, SRF, Sp1 Transcription Factor (SP1) and MEF2A (data not shown).

**Table 4:** Impact of maternal nutrition during mid-to-late gestation on the methylation status of IGF2 DMR2 as assessed by Sequenom Epityper analysis

Amplicon	CpG	Position in amplicon	High diet^a^	Low diet^a^	*P*-value	Correlation with IGF2
			Longissimus dorsi methylation (%)			
IGF2 DMR2 Chr29:51,079,954–51,080,478	1, 2, 3	29, 38, 47	29.1 ± 1.4	27.2 ± 0.9	0.38	*R* = −0.40, *p* = 0.066
	10,11	173, 183	52.3 ± 1.6	48.6 ± 1.3	0.11	*R* = −0.67, *P* = 0.0007
	12, 13, 14	189, 192, 198	40.4 ± 1.5	35.7 ± 0.9	0.019	*R* = −0.70, *P* = 0.0003
	15, 16	202, 206	45.8 ± 1.4	41.9 ± 1.0	0.07	*R* = −0.70, *P* = 0.0003
	20	257	68.1 ± 1.5	63.3 ± 1.4	0.039	*R* = −0.73, *P* = 0.0001
	21, 22	287, 293	48.9 ± 1.4	47.5 ± 0.6	0.44	*R* = −0.57, *P* = 0.0059
	28	384	67.9 ± 2.4	63.0 ± 3.3	0.13	*R* = −0.25, *P* = 0.26
	29	390	89.1 ± 1.8	82.5 ± 1.3	0.014	*R* = −0.64, *P* = 0.0013
	34	482	76.7 ± 4.4	71.6 ± 2.4	0.31	*R* = −0.25, *P* = 0.29
	35	493	68.4 ± 1.0	65.9 ± 0.7	0.073	*R* = −0.71, *P* = 0.0002
	All	na	51.3 ± 1.3	47.4 ± 0.9	0.025	*R* = −0.65, *P* = 0.001
			Semitendinosus methylation (%)			
IGF2 DMR2 Chr29:51,079,954–51,080,478	1, 2, 3	29, 38, 47	31.3 ± 0.8	29.9 ± 0.7	0.28	*R* = −0.66, *P* = 0.0008
	10,11	173, 183	55.0 ± 1.2	51.6 ± 1.0	0.042	*R* = −0.68, *P* = 0.0005
	12, 13, 14	189, 192, 198	41.8 ± 1.3	38.1 ± 0.8	0.06	*R* = −0.78, *P* ≤ 0.0001
	15, 16	202, 206	46.3 ± 0.8	43.7 ± 0.8	0.056	*R* = −0.74, *P* ≤ 0.0001
	20	257	69.4 ± 1.3	66.4 ± 1.1	0.16	*R* = −0.70, *P* = 0.0003
	21, 22	287, 293	50.3 ± 1.2	47.3 ± 1.2	0.11	*R* = −0.49, *P* = 0.019
	28	384	71.7 ± 3.7	61.6 ± 1.9	0.075	*R* = −0.63, *P* = 0.0017
	29	390	87.8 ± 1.5	85.8 ± 1.8	0.46	*R* = −0.44, *P* = 0.038
	34	482	80.1 ± 3.6	75.2 ± 4.1	0.53	*R* = −0.56, *P* = 0.011
	35	493	68.5 ± 1.0	66.6 ± 0.7	0.12	*R* = −0.69, *P* = 0.0004
	All	na	52.8 ± 1.1	49.6 ± 0.9	0.086	*R* = −0.79, *P* ≤ 0.0001

^a^Data represent the methylation percentage for CpG or CpG group and are expressed as lsmeans ± SEM

## Discussion

The influence of maternal nutrition during gestation on fetal programming and more specifically on muscle development has been previously studied in beef cattle [2, 6]. However, our understanding of the molecular mechanisms leading to phenotypic changes in the offspring remains limited. The present study investigated the effect of maternal nutrition during the second half of pregnancy on fetal muscle development and demonstrates that differential nutrient levels can potentially permanently influence the fetus via DNA methylation and gene expression differences even if no obvious differences in phenotypes are observed.

Restricting feed intake to approximately 85% of metabolizable energy requirements had the expected impact on the cow. Restricted cows were lighter at the end of the treatment period because of lower average daily weight gain. Interestingly, the difference in maternal weight gain did not appear to impact fetal growth as fetuses were similar in weight, crown-rump length, and thorax circumference from the HIGH and LOW maternal diet treatments. Our results are in contrast with previous studies in beef cattle that showed that nutritional restriction during the 2nd half of gestation led to lighter birth weights [28, 29]. However, these discrepancies can likely be explained by the milder nutrient restriction imposed in this experiment compared to the more severe nutrient restrictions treatments applied to other ruminant experiments in the literature. On a side note, weaning weights of calves born from cows exposed the same pre-natal dietary treatments as the fetal collection calves were investigated and no differences due to treatment were found (data not shown). Although the authors would like to inform that due to death loss, the presence of twins, and the influences of subsequent research projects which lead to three sex groups among the weaned calves (bulls, steers, and heifers), no concrete conclusions should be made with regard to the weaning data.

Although there may be instances where no apparent fetal phenotypic responses to pre-natal maternal diets are observed, fetuses may show adaptation on the molecular level. In order to assess if the fetuses responded at the molecular level to differential pre-natal nutritional levels, expression of insulin-like growth factors family members was examined in the fetal liver. Our results indicated that restricted fetuses had lower abundance of *IGF1* mRNA, consistent with the hypothesized effect that lower nutrient resources would elicit a metabolic response in the liver of restricted fetuses. Indeed, a study in baboons showed that maternal dietary restriction lead to lower expression of *IGF1* mRNA and protein in fetal liver [30], as well, *IGF1* mRNA was lower in livers of nutrient-restricted fetal sheep when measured at the end of the restriction period [31]. Our results indicate that the fetuses used in this experiment were sensitive to the changes in nutrient supply.

Relative to essential organs such as the heart, skeletal muscle has lower priority for nutrient partitioning in the developing fetus [6]. However, recent work using a KEGG analysis of mRNA-Sequencing results from an experiment that investigated maternal nutrient sources in late gestation on LD mRNA expression in sheep found that energetic metabolism pathways, including insulin signalling, displayed the largest proportion of differentially expressed genes [32]. As such, we evaluated the expression of insulin-like growth factor and myogenic genes in various tissues of HIGH and LOW fetuses. As expected, very few differences were observed in the heart with the exception of a significant increase in *IGF2* in restricted fetuses. We made similar observations in semitendinosus muscle where no significant differences in *IGF2* and *IGF2R* mRNA abundance were detected. On the other hand, increased mRNA abundance of *IGF1*, *IGF1R*, *IGF2R*, *INSR*, *MYOD1* and *MYOG* were observed in the *longissimus dorsi* muscle of restricted fetuses. This is in contrast with a previous study in cattle that showed that a low protein diet during the 1st and/or 2nd trimester altered *IGF1*, *IGF1R*, *IGF2* and *IGF2R* in the ST muscle of restricted animals but not in the LD [33], although several differences exist between the 2 studies including timing of the restriction (early to mid-gestation vs mid- to late gestation), type (total metabolizable energy verses protein) and degree of restriction, and breed of cattle (Angus-Simmental cross-bred vs composite breeds including *Bos indicus*). Differences in gene expression observed between the LD and ST muscle of the fetuses used in the current study suggest that different muscles are affected to varying degrees by nutrient restriction. Just as essential organs have priority over skeletal muscle for nutrient partitioning, it is possible that muscles with different functions, for example postural vs locomotion, also have different priority for nutrient partitioning. This hypothesis is attractive as muscle with different functions may also differ in muscle fiber number, size and composition [34], which would be reflected in differential gene expression.

It is interesting to note that all differentially expressed genes in LD muscle have greater abundance in the muscle of LOW fetuses. This led us to hypothesize that muscle development in LOW fetuses was delayed as compared to development in HIGH fetuses, but also that the muscle of the HIGH diet fetuses had transitioned from myogenesis to adipogenesis by the time of sample collection. To address the latter hypothesis, we designed primers for *PPARG* and CCAAT/Enhancer Binding Protein Alpha (*CEPBA*) which are known inducers of adipogenesis [35]. Using two different sets of primers we could not successfully amplify *CEPBA* in LD and ST muscle. However, *PPARG* mRNA abundance was higher in the LD muscle of LOW fetuses suggesting that the fetuses fed the HIGH diet had not increased adipogenesis. Further investigation into the literature revealed potential roles for *PPARG* expression in skeletal muscle regulating its internal lipid metabolism [36]. Increased expression of *PPARG* in skeletal muscle fibres in offspring has been shown to be associated with pre-natal consumption of a maternal cafeteria diet in rats [16]. Although no direct evidence exists linking pre-natal maternal undernutrition to increased *PPARG* expression in skeletal muscle, other groups have hypothesized that mobilization of fatty acids in the maternal circulation from maternal adipose reserves, brought about by maternal undernutrition, might elicit similar responses in *PPARG* expression in fetal muscle as is seen with the maternal overnutrition model [37]. Therefore higher expression of *PPARG* in LOW diet fetuses might be an appropriate response to the nutritional treatment applied to their dams in the present experiment.

MicroRNAs have been shown to be important for various biological processes including muscle development [38, 39]. The miRNA miR-1 and miR-133a are known to modulate proliferation and differentiation in skeletal muscle [38]. Their expression is also modulated by myogenic transcription factors including MEF2A, SRF and MYOD1. Our results revealed that miR-1 and miR-133a expression is increased in the *longissimus dorsi* muscle of the HIGH diet fetuses. Interestingly, in human fetal skeletal muscle, miR-1 and miR-133a expression were found to increase during the late stages of development [39]. This strengthened our previous hypothesis suggesting that the LD muscle of the LOW diet fetuses are developmentally delayed compared to that of the HIGH fetuses.

Our results suggest that differential pre-natal nutrient level during the 2nd half of pregnancy can alter the methylation status of *IGF2* as shown by the difference in methylation of several CpG units in the DMR2 of *IGF2*. This observation is curious since no differences in *IGF2* mRNA abundance were observed in LD and ST muscles. However, the standard error for *IGF2* mRNA expression is relatively large suggesting a wide range of response between fetuses belonging to the same treatment. Correlation analysis indicate a negative relationship between *IGF2* mRNA abundance and CpG methylation in DMR2, supporting our hypothesis that increased methylation reduces *IGF2* expression. This is in contrast with the suggested mechanism for *IGF2* expression since the DMR2 is known to contain a methylation sensitive activator that increases *IGF2* expression with increasing methylation [40]. Analysis of our DMR2 partial sequence with the web-based prediction program TFBIND revealed several predicted transcription factor binding sites, including MYOD1, MEF2A, SRF and SP1 that are known regulators of myogenesis. It is therefore tempting to speculate that increased methylation in that region altered transcription factor or enhancer binding, leading to reduced gene expression. *IGF2* and *MYOD1* are known to mutually modulate their expression in muscle [41]. Therefore the positive correlation observed between *IGF2* and *MYOD1* mRNA abundance certainly reinforces our hypothesis.

In the current study, we also examined the expression of growth, angiogenic, and solute carrier family members in the uterus and placenta of animals exposed to HIGH and LOW diets. Our observations reveal no significant differences in gene expression in the caruncle and cotyledon suggesting that the placentome of these cattle was not altered by the nutritional treatment at the time of tissue collection. This is also corroborated by the lack of differences in placental and uterine weight, fetal weight, crown-rump length, and thorax circumference observed between the HIGH and LOW fetuses. Our results are in contrast with previous studies that have shown that nutritional restriction during the 1st and 2nd half of pregnancy can be detrimental to placental development. In cattle, nutritional restriction from day 30 to 125 of gestation was shown to decrease placentome weight [41, 42], while in the ewe, maternal undernutrition during early- to mid-gestation led to increased placental weight [43]. On the other hand, nutrient restriction during the 2nd half of gestation in rats has been shown to decrease the placental:fetal weight ratio [44]. Maternal undernutrition can also alter placental vascularity leading to reduction in blood flow and consequently affecting nutrient transfer to the fetus. This was shown in cattle where nutrient restriction during early- to mid-gestation affected placental vascularity and expression of *VEGFA* mRNA [42, 45]. In cows, development of the placentome is established in early to mid-gestation and therefore the impact of a mild nutrient restriction during mid-to-late gestation is likely to have limited impacts on the uterus and placenta [1, 46]. Therefore, the changes in gene expression observed in the muscle in the current study are unlikely to originate from major alterations in placental development or gene expression related to nutrient transfer to the fetus.

## Conclusions

The findings of the present study demonstrate that differential pre-natal nutritional level during the 2nd half of gestation can have impacts on fetal development even if no obvious phenotypic differences are observed in late term fetuses. This work illustrates the importance of maternal nutrition during gestation for cattle, even when nutrition restriction is mild. However, further research is needed to determine if these differences in expression in growth-factor-related genes, and genes involved in muscle development in the fetus, translate to observable phenotypic differences later in life. It would be particularly important if DNA methylation and gene expression differences result in changes in growth performance, feed efficiency, and carcass characteristics. Our data also suggests that different muscle groups respond differently to nutrient insults, likely as a consequence of their function and muscle fiber type composition. Further research is needed to determine if the changes in DNA methylation observed in the DMR2 of *IGF2* are transient or are heritable. Using fetal programming as a management tool for beef producers may provide opportunities for improvements for growth and production traits in cattle.

## Abbreviations

*ACTB*: Beta-Actin
ADG: average daily gain
*CEPBA*: CCAAT/Enhancer Binding Protein Alpha
DMI: dry matter intake
DMR2: Differentially Methylated Region 2
*EEF1A2*: Eukaryotic Translation Elongation Factor 1 Alpha 2
*FLT1*: Fms Related Tyrosine Kinase 1
*GAPDH*: Glyceraldehyde-3-Phosphate Dehydrogenase
gDNA: Genomic DNA
HIGH: cows fed to ad-libitum intake (~140% of metabolizable energy requirements (NRC, 1996)) during the second half of gestation
*HMBS*: Hydroxymethylbilane Synthase
ICR: intergenic control region
*IGF1*: Insulin Like Growth Factor 1
*IGF1R*: Insulin Like Growth Factor 1 Receptor
*IGF2*: Insulin Like Growth Factor 2
*IGF2R*: Insulin Like Growth Factor 2 Receptor
*INSR*: Insulin Receptor
*KDR*: *Kinase Insert Domain Receptor*
LD: *longissimus dorsi*
LOW: cows restricted to 85% of metabolizable energy requirements (NRC, 1996) during the second half of gestation
LSD: Least Significant Difference
*MEF2A*: Myocyte Enhancer Factor 2A
miR-1: microRNA 1
miR-133a: microRNA 133a
miR-16b: microRNA 16b
miR-191: microRNA-191
miRNA: micro RNA
MRF: myogenic regulatory factors
*MYOD1*: Myogenic Differentiation 1
*MYOG*: Myogenin
*PGF*: Placental Growth Factor
*PPARG*: Peroxisome Proliferator Activated Receptor Gamma
*PPIA*: Peptidylprolyl Isomerase A
qPCR: quantitative PCR
RIN: RNA Integrity Number
RNU6B: RNA U6 Small Nuclear 6
RT: reverse transcription
*SLC2A1*: Solute Carrier Family 2 Member 1
*SLC2A3*: Solute Carrier Family 2 Member 3
*SLC7A1*: Solute Carrier Family 7 Member 1
SP1: Sp1 Transcription Factor
*SRF*: *Serum Response Factor*
ST: *semitendinosus*
*VEGFA*: Vascular Endothelial Growth Factor A
*YWHAZ*: Tyrosine 3-Monooxygenase/Tryptophan
